# Simulation Analysis on the Fracture Failure of S2 Alloy Steel Screwdriver Bits

**DOI:** 10.3390/ma19163443

**Published:** 2026-08-14

**Authors:** Xindi Feng, Zhongjun Wang

**Affiliations:** School of Materials and Metallurgy, University of Science and Technology Liaoning, Anshan 114051, China

**Keywords:** S2 alloy steel, screwdriver bits, numerical simulation, heat treatment, fracture failure, carbon potential

## Abstract

**Highlights:**

- Identified the synergistic failure mechanism of S2 steel screwdriver bits, revealing that non-planar fractures result from the combined effects of surface decarburization, non-uniform martensitic microstructure, and multi-level stress coupling.
- Developed an integrated quantitative analysis approach using Deform-3D quenching simulation and Ansys Workbench torsion simulation to systematically analyze microstructure evolution, residual stress fields, and working stress concentration.
- Revealed the dual control mechanism of quenching temperature and carbon potential: insufficient austenitizing at 830 °C produces non-uniform martensite, while low carbon potential below 0.40 causes surface decarburization. Both factors independently and synergistically reduce the qualification rate.
- Optimized heat-treatment process with precise carbon potential control (0.40–0.45) and parameters (860 °C austenitizing, 170 °C tempering) to achieve uniform tempered martensite, enabling stable planar fracture and a 100% qualification rate.

**Abstract:**

The microstructures and torsional fracture morphologies of S2 alloy steel screwdriver bits were characterized by scanning electron microscopy (SEM). The bits were oil-quenched from 830 °C and 860 °C and subsequently tempered at 150 °C and 170 °C under three carbon potential levels (0.35, 0.40, and 0.45). In parallel, Deform-3D and Ansys Workbench were employed to simulate and compare the microstructure evolution during quenching, the residual stress field after quenching and tempering, and the stress distribution developed under torsional loading. The results reveal that the non-planar fracture and low qualification rate of the bits arise from two independent but synergistic mechanisms: (1) insufficient austenitizing at 830 °C fails to produce fully uniform austenite, resulting in non-uniform martensitic microstructure and inhomogeneous hardness distribution after quenching; (2) low furnace carbon potential (≤0.35) causes surface decarburization and the formation of massive ferrite at the near-surface region, which acts as preferential crack initiation sites. Furthermore, the transformation stress generated during quenching, the residual stress remaining after tempering, and the stress concentration at tooth edges under service loading jointly promote crack initiation and propagation. A uniform, high-hardness tempered martensite microstructure is obtained when the bits are austenitized at 860 °C with the carbon potential strictly maintained between 0.40 and 0.45, held for 60 min before oil quenching, and air-cooled after tempering at 170 °C. This optimized heat-treatment route eliminates surface decarburization, ensures microstructural homogeneity, reduces residual stress, and enables the bits to fail by planar fracture under torsional load with 100% qualification rate.

## 1. Introduction

Electric screwdrivers are ubiquitous power tools that tighten and loosen threaded fasteners through interchangeable screwdriver bits. With the continuous advancement of high-end manufacturing and precision-assembly industries, such tools have been deployed extensively in machinery manufacturing, medical-device production, automotive assembly, aerospace fastener installation, and consumer-electronics fabrication. As the core consumable component that directly bears the working load, the screwdriver bit governs assembly efficiency, operational safety, and production consistency. Consequently, the structural optimization, material upgrading, and failure-mechanism analysis of screwdriver bits have long been important topics in industrial engineering, attracting sustained attention from both academic researchers and manufacturing engineers.

In recent years, considerable effort has been devoted to improving screwdriver systems from several perspectives. Duryat systematically described the material design and screening workflow for screwdriver bodies by combining the material-index method with a multi-criteria evaluation matrix, providing a quantitative framework for material selection [1]. To address the overload failure of screwdriver bits caused by severe stress concentration at the contact interface, Tong and Yang derived four optimized bit profiles from involute mathematical theory and numerical algorithms, effectively relieving the local stress peaks [2]. Nieoczym et al. quantitatively characterized the kinetic-energy evolution of both the driving modules and the executive components of electric screwdrivers across different operating stages and proposed targeted strategies for reducing energy consumption [3]. Overall, existing studies focus predominantly on structural innovation, material-selection methodology, and energy efficiency, whereas systematic investigations of the fracture-failure mechanism of screwdriver bits under realistic torsional service conditions remain relatively scarce.

Compared with conventional candidate materials for industrial screwdriver bits—including Grade 45 medium-carbon structural steel, Cr–Mo alloy steel, and 50CrV alloy steel—S2 impact-resistant tool steel, as specified by ASTM standards, offers a superior combination of hardness, impact toughness, and wear resistance, making it the preferred material for high-performance heavy-duty bits [4]. After standard quenching and tempering, the matrix of S2 steel is dominated by fine tempered martensite; this microstructure provides the high hardness and wear resistance required for heavy-duty service, consistent with heat-treatment responses observed across high-carbon tool steels [5]. However, S2 steel is highly sensitive to its thermal-processing parameters: unstable cooling rates during the hot rolling of wire rods cause pronounced fluctuations in phase fractions and carbide distribution, which in turn produce severe inconsistency in the as-received mechanical properties and pose inherent quality risks for the finished product [6].

During the cold-forming and machining stages, the cutting parameters, tool wear, and running stability of integrated turning–milling machines exert a decisive influence on the surface integrity of the finished bit, including its surface roughness, residual state, and subsurface deformation [4]. From a metallurgical standpoint, the silicon and molybdenum in S2 steel render the material highly susceptible to high-temperature oxidation and surface decarburization during heat treatment [7]. A decarburized surface layer markedly lowers the surface hardness, weakens the wear resistance, and introduces unfavorable tensile residual stress, thereby providing preferential sites for fatigue-crack initiation and propagation [8]. In addition, improper control of the austenitizing temperature and the furnace carbon potential can further aggravate microstructural inhomogeneity and degrade the overall mechanical performance of the product [6].

In mass production and long-term service, screwdriver bits that carry inherent manufacturing defects—such as surface decarburization, unfavorable residual stress, and geometric stress concentration—are prone to abnormal non-planar fracture under the coupled action of torsional shear, abrasive wear, and intermittent impact. Unlike normal planar fracture, which exhibits predictable ductile behavior, non-planar fracture often occurs abruptly and without obvious macroscopic plastic deformation; this not only drastically shortens the fatigue life of the component but also introduces serious safety hazards in high-strength assembly scenarios. To date, there is still a lack of systematic studies clarifying the dominant factors that govern the non-planar fracture of S2 steel screwdriver bits, and the correlation between the evolution of the internal stress field under torsional loading and the actual fracture path has not been thoroughly elucidated. The underlying failure mechanism under the process fluctuations typical of mass production also remains to be revealed.

In the present work, comprehensive experimental characterization—including fractographic observation, metallographic analysis, and inspection of internal non-metallic inclusions—was performed on S2 steel screwdriver bits treated at two quenching temperatures (830 °C and 860 °C) and two tempering temperatures (150 °C and 170 °C). Combined with numerical simulation of the residual stress field developed throughout the heat-treatment process (Deform-3D) and of the stress distribution under torsional loading (Ansys Workbench), the root causes of the high incidence of non-planar fracture detected during mass-production inspection were interpreted systematically from both microstructural and mechanical perspectives. On this basis, an optimized heat-treatment scheme was proposed to obtain uniform, high-hardness tempered martensite and to ensure a stable planar fracture mode. The findings provide theoretical guidance and technical support for the process optimization and quality control of high-performance S2 screwdriver bits in industrial mass production.

## 2. Experimental Materials and Methods

### 2.1. Experimental Materials

Commercial PH2 Phillips-head screwdriver bits manufactured from ASTM-grade S2 shock-resistant tool steel from Liaoning Keledi Light Industry Co., Ltd. in Chaoyang, China were chosen as the research material. The nominal chemical composition of the as-received S2 steel is listed in Table 1.

All specimens were pre-machined to their final geometric dimensions by cold heading and turning–milling, yielding a uniform surface finish free of macroscopic machining defects such as burrs and edge cracks prior to heat treatment.

According to the technical specifications for industrial heavy-duty screwdriver bits, qualified S2 bits must satisfy two core requirements after final heat treatment: first, the Rockwell hardness of the matrix must be no less than 60 HRC to guarantee adequate wear resistance and load-bearing capacity; second, the minimum static torsional fracture torque must reach 10 N·m to resist torsional overload during repeated assembly.

### 2.2. Heat-Treatment Procedure

All heat-treatment experiments were conducted in an RXQ-550-12 multifunctional controlled-atmosphere pit furnace (Wuhan Hankou Furnace Co., Ltd., Wuhan, China). Methanol was dripped continuously into the chamber as the atmosphere carrier, and the furnace carbon potential was regulated precisely at three levels (0.35, 0.40, and 0.45) to explore the influence of surface carbon content on the fracture behavior of the bits.

**Figure 1:**
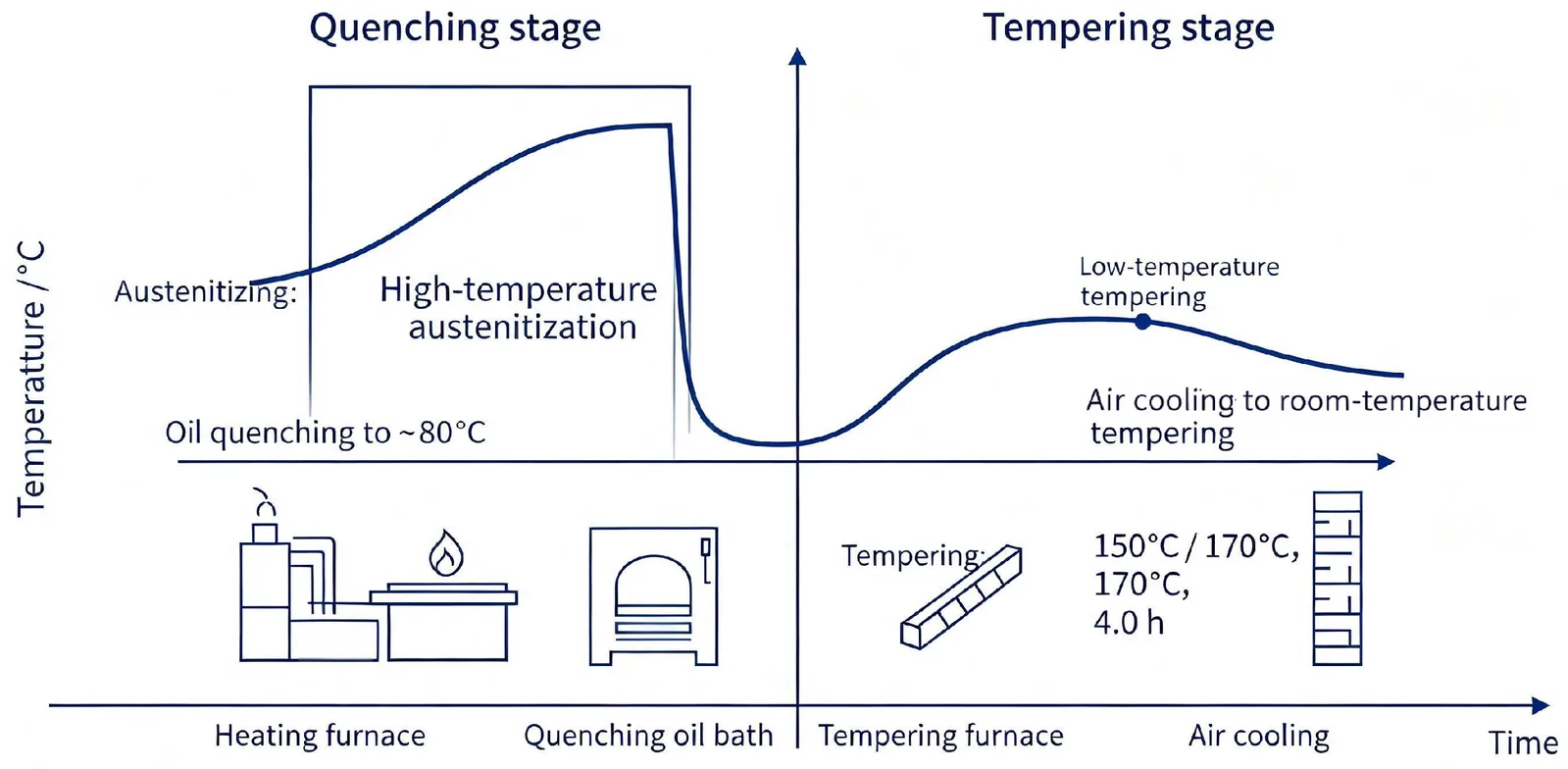
Schematic illustration of the two-stage heat-treatment process including high-temperature austenitizing–quenching and low-temperature tempering for S2 steel screwdriver bits.

A full-factorial experimental design was adopted to investigate the effects of austenitizing temperature and tempering temperature on the microstructure and mechanical performance of the specimens. The schematic diagram of the overall quenching and tempering heat-treatment cycle is shown in Figure 1. Two austenitizing temperatures (830 °C and 860 °C) were selected within the full-austenitization range of S2 steel. After an isothermal hold of 60 min at the target temperature—sufficient to ensure uniform austenitization and adequate dissolution of carbon into the austenite—the specimens were transferred immediately into commercial quenching oil. Quenching was terminated when the specimen temperature fell to approximately 80 °C, so as to obtain a fully martensitic microstructure while avoiding excessive thermal stress and the risk of quench cracking.

The quenched specimens were subsequently tempered in an RJ2-55-6 pit tempering furnace (Wuhan Hankou Furnace Co., Ltd., Wuhan, China) at two temperatures (150 °C and 170 °C) for a fixed holding time of 4.0 h. This low-temperature tempering was designed to relieve quenching residual stress and improve matrix toughness while preserving the high hardness of the martensite. Four parallel specimens were prepared for each heat-treatment regime to ensure repeatability and statistical reliability. After tempering, all specimens were air-cooled to room temperature and then sandblasted to remove the surface oxide scale, followed by anti-rust oil sealing, in full agreement with the post-treatment practice used in industrial mass production.

### 2.3. Hardness Test, Torsional Fracture Test and Qualification Criterion

Hardness tests were carried out using an HR-150D Rockwell hardness tester (Shanghai Juhui Instrument Manufacturing Co., Ltd., Shanghai, China) with the Rockwell C (HRC) scale, which features an effective measuring range of 20–70 HRC and fully accommodates the high hardness (50–70 HRC) of the heat-treated S2 alloy steel screwdriver bits. Indentations were positioned on the flat prismatic surfaces of the bits, with a preliminary test force of 98 N, a total test force of 1471 N, and a full-load dwell time of 4 s.

**Figure 2:**
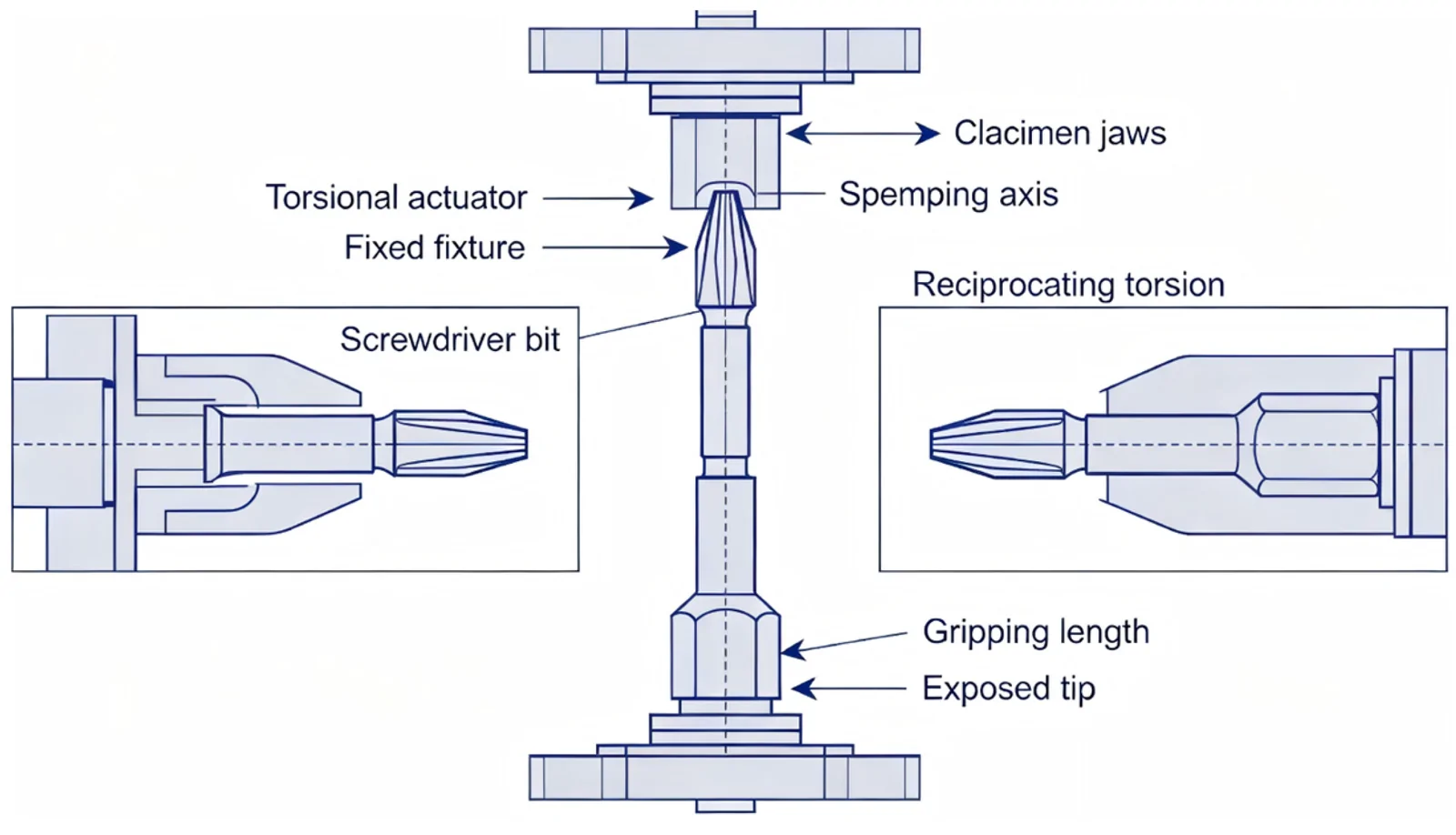
Schematic illustration of the fixture clamping setup for PH2 screwdriver bit specimens during cyclic torsional fracture testing.

Reciprocating cyclic torsional fracture tests were performed on a RUMUL high-frequency fatigue testing machine to reproduce the repeated loading experienced by screwdriver bits in service. The clamping configuration of screwdriver bit specimens on the torsional fracture testing machine is illustrated in Figure 2. The shank of each PH2 screwdriver bit was firmly gripped by the testing machine fixtures to guarantee coaxiality under alternating torsional loading, and the bit tip was kept free to simulate practical working conditions. The machine operates within a frequency range of 40–260 Hz, with static and dynamic load measurement accuracy better than a ±0.5% full scale. The torsional load was controlled under load mode, and symmetrical cyclic torsion loading (stress ratio R = −1) was adopted to simulate alternating twisting loads during practical service. Fully reversed torsional loading (stress ratio R = −1) was applied to all specimens until complete fracture. The peak cyclic torque was set to 8 N·m—80% of the specified minimum static torsional fracture torque—to evaluate the torsional reliability of the bits under long-term cyclic service.

**Figure 3:**
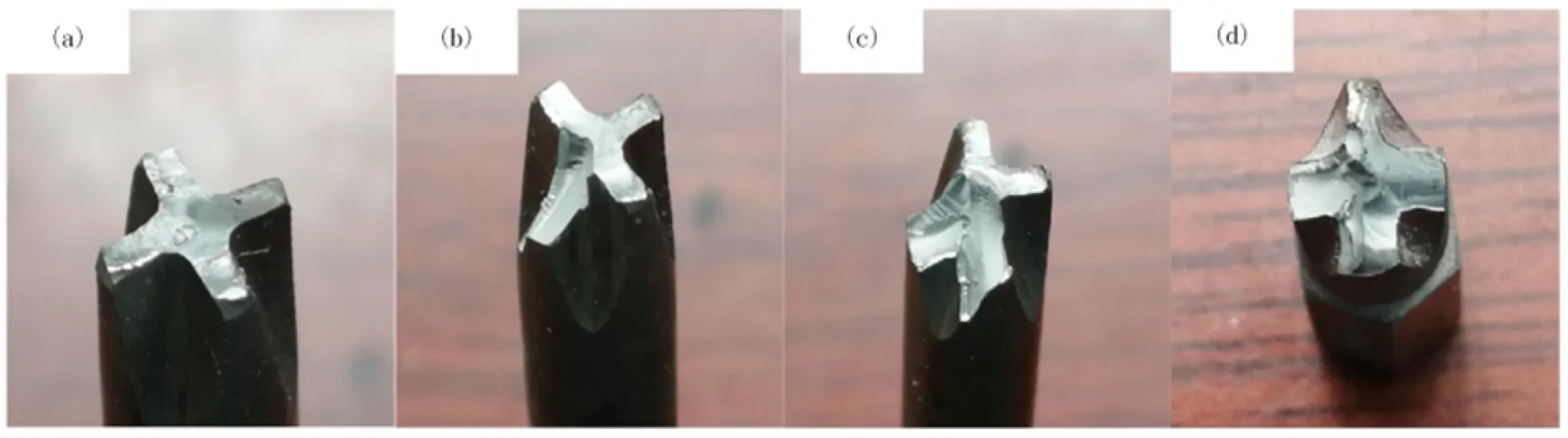
Qualification criteria of screwdriver bits: (**a**) qualified specimen; (**b**) Type-A unqualified specimen; (**c**) Type-B unqualified specimen; (**d**) Type-C unqualified specimen.

After the test, the fracture morphology of each specimen was classified according to the failure mode of its four working teeth. The qualification criterion is illustrated schematically in Figure 3: a specimen is deemed qualified only when all four teeth on the fracture surface exhibit typical planar fracture features. Planar fracture generally reflects good matrix toughness and stable failure behavior, whereas abnormal non-planar fracture indicates a brittle tendency and poor service reliability and is therefore judged unqualified.

### 2.4. Microstructure and Fractographic Observation

Microstructures and torsional fracture morphologies were characterized using a Philips XL30W/TMP scanning electron microscope (Philips Electron Optics, Eindhoven, The Netherlands) operated in secondary electron mode at an accelerating voltage of 20 kV. Systematic examinations were conducted on two sets of regions: the cross-sectional center of S2 alloy steel screwdriver bits for microstructure analysis and typical fracture surface zones (the tooth-root stress concentration zone, central matrix region, and surface edge) for fractographic characterization. This enabled the identification of crack initiation sites, crack propagation paths, and typical fracture features including cleavage planes, quasi-cleavage facets, tear ridges, and secondary cracks [9]. Based on these fractographic results, fracture modes under different heat-treatment conditions were distinguished, and the underlying failure mechanisms were further elucidated. Metallographic specimens were prepared via the conventional hot mounting method and etched with 8% Nital solution.

### 2.5. Numerical Simulation

SolidWorks 2024 SP5 three-dimensional modeling software (Dassault Systèmes SolidWorks Corporation, Waltham, MA, USA) was adopted to build the solid model of the PH2 Phillips screwdriver bit. A one-quarter symmetric model was intercepted for finite element calculation. This approach reduces the total mesh quantity and facilitates post-processing observation of cross-sectional results including temperature distribution, phase transformation and stress field. The working end of the screwdriver bit is the core research region, so local mesh refinement was implemented on this zone. After refinement, the total number of meshes reaches 180,000.

A precise three-dimensional model of the PH2 screwdriver bit was constructed from its actual structural dimensions. The core load-bearing structures—the cross-shaped working head and the cylindrical shank—were retained in full, while minor non-functional features such as small edge chamfers were simplified to reduce computational cost without compromising accuracy. Two sets of simulations were then performed, addressing the heat-treatment process and the torsional-loading behavior, respectively.

Deform-3D V11.2 (Scientific Forming Technologies Corporation, Columbus, OH, USA), which is specialized for metal thermal-processing simulation [10,11], was used to compute the microstructure and residual stress fields of the bit after quenching and tempering. The model was discretized with tetrahedral elements, and local mesh refinement was applied to the surface layer and the tooth-root regions, where steep stress and temperature gradients occur. The thermophysical parameters of S2 steel—thermal conductivity, specific heat capacity, latent heat of phase transformation, and the thermal-expansion coefficient—were taken from the software’s built-in material database and corrected to the actual chemical composition. The Koistinen–Marburger (K-M) model was introduced to describe the martensitic transformation kinetics, and the heat-transfer boundary conditions for oil quenching and tempering air cooling were set consistently with the experimental parameters [12,13]. The simulation outputs comprised the volume-fraction distribution of tempered martensite, the magnitude and distribution of the residual stress, and the microhardness profile across the cross-section.

Ansys Workbench (https://ansys.synopsys.com/products/ansys-workbench (accessed on 29 June 2026)) was then employed to analyze the torsional mechanical response of the bit under service conditions. The static-structural module was used, and the residual stress field obtained from the Deform-3D heat-treatment simulation was imported as the initial stress state to achieve a multi-field coupled analysis. The mechanical properties of the heat-treated S2 steel—elastic modulus, Poisson’s ratio, and yield strength—were defined as material attributes. One end of the shank was fully fixed, while a rated torque was applied to the working end, consistent with the loading in the torsion test. A hexahedral-dominant mesh with local refinement at the tooth root was adopted to ensure accuracy [14]. The equivalent stress distribution, the peak stress location, and the stress-concentration coefficient under torsional load were extracted to provide a mechanical basis for explaining the fracture-initiation position and failure mode observed experimentally.

## 3. Result and Discussion

### 3.1. Microstructure Evolution and the Effect of Quenching Temperature

**Figure 4:**
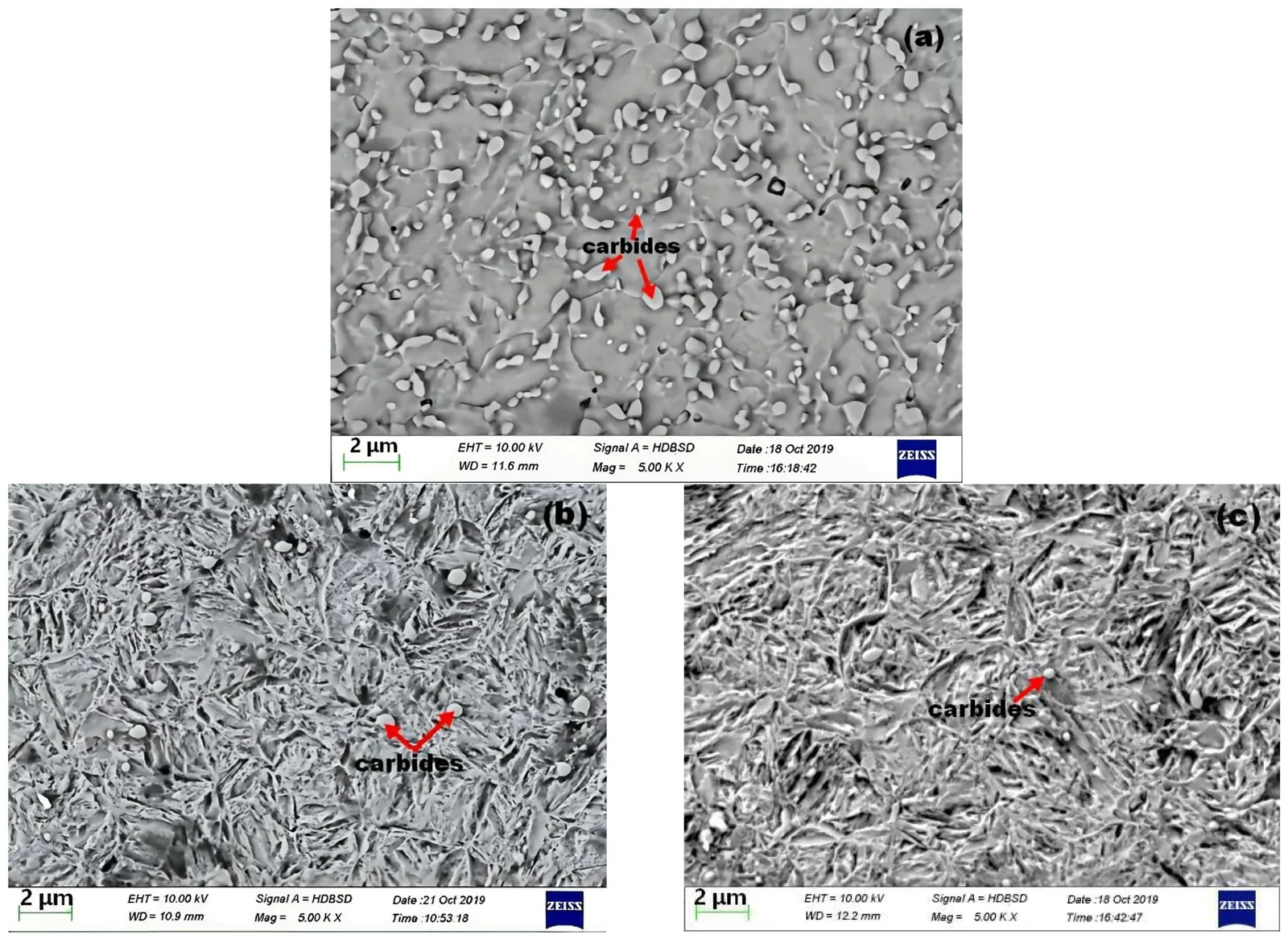
Microstructures before and after quenching and tempering: (**a**) before quenching; (**b**) quenched at 830 °C for 6 h and tempered at 150 °C for 4 h; (**c**) quenched at 860 °C for 6 h and tempered at 170 °C for 4 h.

Figure 4 shows the microstructures of S2 alloy steel screwdriver bits before and after quenching and tempering. As seen in Figure 4a, the as-received microstructure before quenching contains a large number of carbide particles with non-uniform size distribution, with maximum sizes ranging from 0.5 to 1.5 μm. These coarse and unevenly distributed carbides are the inherent microstructural feature of the hot-rolled wire rod.

After quenching at 830 °C for 60 min followed by tempering at 150 °C for 4 h, the microstructure transforms to tempered martensite, but a considerable amount of undissolved carbide particles remains, with maximum sizes still reaching approximately 1.0 μm (Figure 4b). More importantly, the martensitic matrix itself exhibits obvious non-uniformity at this quenching temperature. This observation is further confirmed by the Deform simulation results presented in Section 3.3.

When the quenching temperature is raised to 860 °C (60 min holding, followed by tempering at 170 °C for 4 h), the microstructure becomes uniform tempered martensite. Both the quantity and maximum size of residual carbides decrease markedly compared with the 830 °C quenching route, with the largest carbide particles measuring less than 0.5 μm (Figure 4c). The carbide distribution is also more homogeneous throughout the matrix.

The microstructural difference between the two quenching temperatures is the first fundamental factor governing the final qualification rate. At 830 °C, the austenitizing temperature is insufficient for complete dissolution of alloy carbides and full homogenization of austenite composition. The non-uniform austenite directly transforms into non-uniform martensite during quenching, which in turn leads to inhomogeneous hardness distribution across the bit cross-section. Regions with lower martensite content or coarser martensite laths exhibit lower hardness and weaker shear resistance, creating mechanical “soft spots” in the otherwise hard matrix. Under torsional loading, these soft spots preferentially undergo plastic deformation and act as crack initiation sites, ultimately leading to non-planar fracture and a low qualification rate. In contrast, austenitizing at 860 °C ensures sufficient carbide dissolution and uniform austenite composition, which transforms into uniform martensite during quenching and provides consistent hardness and mechanical properties throughout the component.

### 3.2. Surface Decarburization and the Effect of Carbon Potential

**Figure 5:**
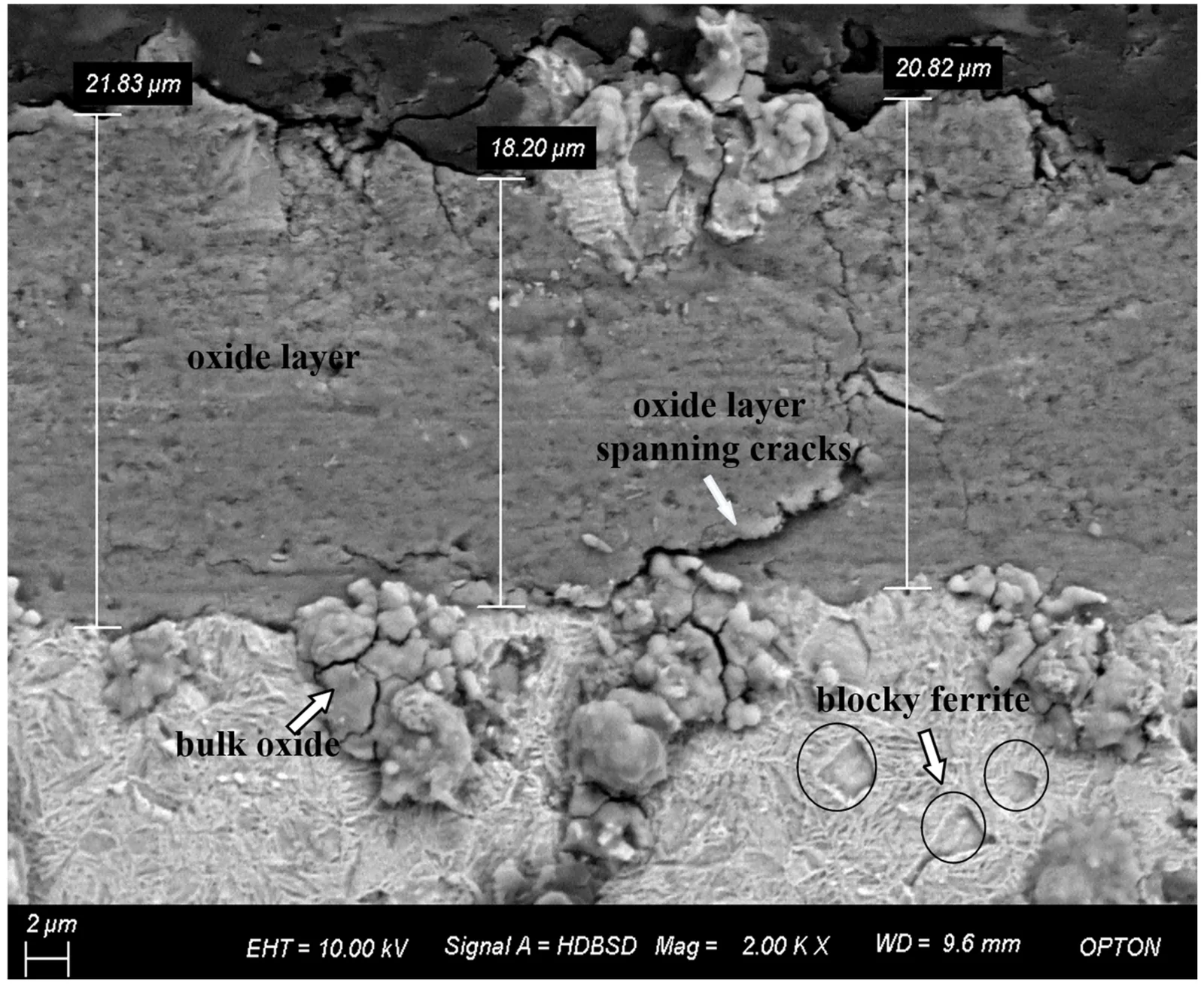
Microscopic morphology near the tooth edge of the fracture, showing some ferrite formed by surface decarburization.

Figure 5 shows the subsurface microstructure adjacent to the tooth-edge fracture of a non-planar specimen treated at 860 °C quenching, 170 °C tempering, and a relatively low furnace carbon potential of 0.35. The matrix is predominantly tempered martensite, but a small quantity of massive ferrite grains is distributed in the near-surface region close to the tooth flank. Because this combination of quenching and tempering temperatures (860 °C + 170 °C) has been proven to produce uniform bulk martensite (as demonstrated in Section 3.1 and Section 3.3), the appearance of free ferrite at the subsurface can be attributed solely to the low carbon potential, and it directly confirms the occurrence of surface decarburization during high-temperature heat treatment.

S2 alloy steel has a high matrix carbon content (0.45–0.55 wt%), which raises the chemical activity of carbon atoms at elevated temperature. During the austenitizing hold, when the furnace carbon potential is lower than the equilibrium carbon potential of the matrix (approximately 0.40 for S2 steel at 860 °C), surface carbon diffuses outward and reacts with oxidizing species such as CO_2_ and H_2_O to form CO, causing continuous loss of surface carbon. The higher the initial carbon content of the steel, the larger the concentration driving force for outward carbon diffusion, which aggravates decarburization and deepens the decarburized layer [15]. At a carbon potential of 0.30–0.35, this decarburization effect is particularly severe, resulting in the formation of a continuous ferrite layer at the part surface.

The formation of massive ferrite through decarburization exerts multiple detrimental effects on the torsional performance.

First, compared with the high-strength tempered martensite matrix (hardness > 60 HRC), the massive ferrite has much lower hardness (approximately 150–200 HV, equivalent to <15 HRC) and shear strength. This creates an extreme mechanical property mismatch at the surface. Under cyclic torsion, the property mismatch between soft ferrite and hard martensite causes uncoordinated plastic deformation at the phase interface, inducing pronounced local stress concentration. Fatigue cracks therefore nucleate preferentially within the soft ferrite or at the ferrite–martensite boundary.

Second, the decarburized surface layer introduces tensile residual stress after quenching due to the volume change difference between ferrite and martensite during cooling. This tensile residual stress superimposes on the applied torsional stress, further promoting crack initiation and early propagation.

Third, in addition to decarburization, a brittle oxide layer forms on the outermost surface under the high-temperature oxidizing atmosphere. Under the combined action of thermal stress during quench cooling and transformation stress from martensitic transformation, through-thickness cracks readily initiate and propagate across the oxide layer. These surface cracks provide channels for further inward diffusion of oxidizing species [16].

**Figure 6:**
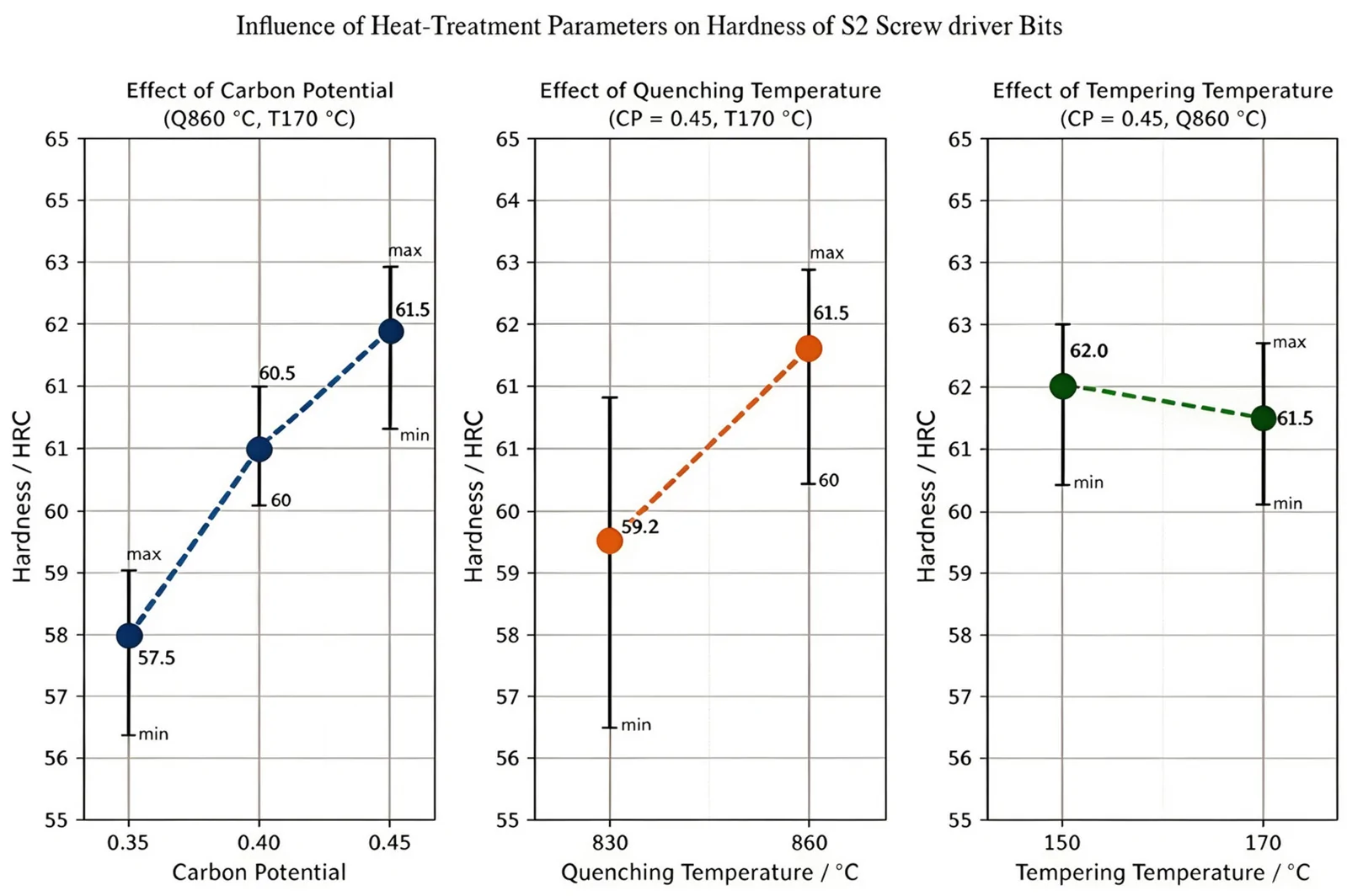
Effects of carbon potential, quenching temperature and tempering temperature on the hardness of S2 alloy steel screwdriver bits. The carbon potential test was performed at a quenching temperature of 860 °C and tempering temperature of 170 °C; the quenching temperature and tempering temperature tests were conducted with a fixed carbon potential of 0.45. Vertical error bars denote the maximum and minimum hardness data.

The low-hardness massive ferrite, the brittle surface oxide layer, and the associated tensile residual stress jointly degrade the fatigue strength and microstructural homogeneity of the surface layer. This explains why, even at the optimal quenching and tempering temperatures (860 °C + 170 °C), the qualification rate drops to only 50% when carbon potential is as low as 0.35 (Figure 6). When carbon potential is controlled at 0.40–0.45, the furnace potential is essentially equal to or slightly higher than the equilibrium carbon potential of S2 steel, which effectively suppresses outward carbon diffusion, prevents the formation of massive surface ferrite, and ensures uniform surface microstructure and mechanical properties.

Figure 6 presents the effects of carbon potential, quenching temperature, and tempering temperature on the hardness of S2 alloy steel screwdriver bits. It can be seen that hardness generally increases with increasing quenching temperature and tempering temperature within the tested range. The carbon potential test, conducted at the optimal 860 °C quenching and 170 °C tempering condition, confirms that sufficient carbon potential is required to maintain surface hardness above 60 HRC.

### 3.3. Fracture Morphology and Qualification Rate Analysis

**Figure 7:**
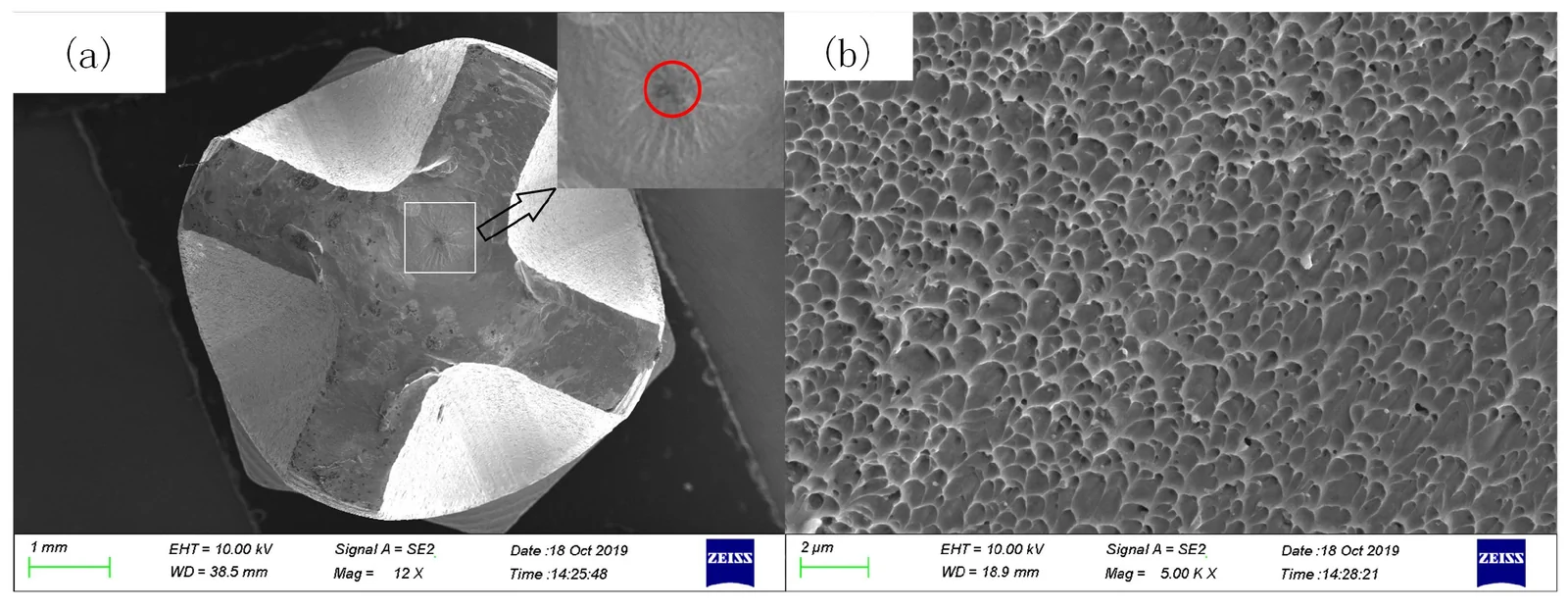
Planar fatigue fracture morphology of an S2 alloy steel screwdriver bit: (**a**) macroscopic fracture appearance; (**b**) microscopic morphology of shear dimples on the fracture surface.

Figure 7 shows the typical torsional fracture characteristics of S2 alloy steel bits oil-quenched at 860 °C and tempered at 170 °C, which corresponds to the optimized process with a 100% qualification rate. This group exhibits a typical planar fracture mode, consistent with the qualified-failure criterion defined above. At the macroscopic scale (Figure 7a), the entire fracture surface is smooth and flat and lies essentially perpendicular to the axis of the bit, with no obvious oblique shear lip or large-scale plastic deformation at the edge. A distinct fatigue-crack initiation site is clearly visible near the subsurface of the tooth root, which is the region of most severe stress concentration under torsional loading. Taking the crack origin as the center, well-developed radial ridges and river-like striations radiate outward toward the edge and the center of the cross-section.

At higher magnification (Figure 7b), the central region of the fracture surface is covered by numerous uniformly distributed shear dimples 0.2–0.8 μm in diameter. Pronounced tear ridges separate adjacent dimples, forming a typical torsional-fracture morphology of shear dimples separated by tear ridges. The abundant dimples and well-developed tear ridges together indicate that the fracture is dominated by microvoid coalescence, a typical feature of ductile fracture [17].

**Figure 8:**
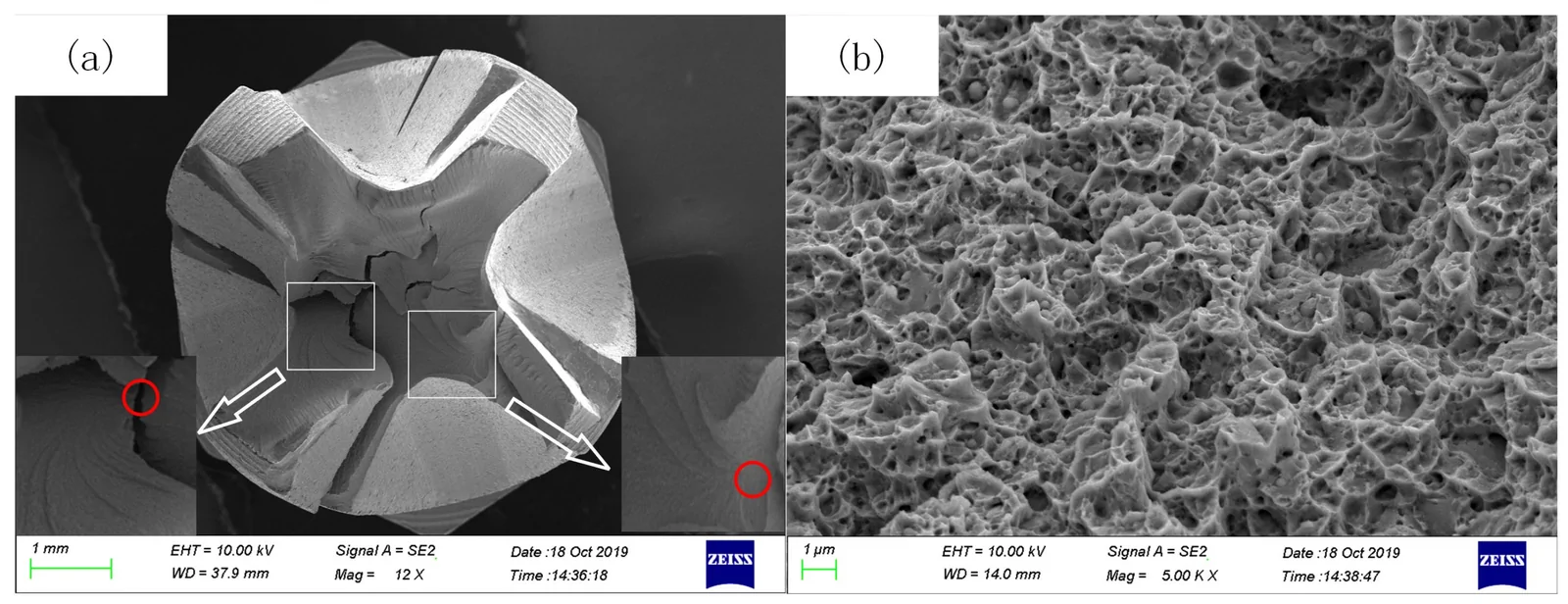
Non-planar fracture morphology of a screwdriver bit quenched at 860 °C and tempered at 150 °C: (**a**) macroscopic fracture surface; (**b**) microscopic fracture morphology showing mixed ductile–brittle features.

In contrast, bits that fail by non-planar fracture show markedly different fractographic features; these specimens are found predominantly in process groups with insufficient quenching temperature, low carbon potential, or inadequate tempering. As shown in Figure 8a, the fracture surface of a non-planar specimen forms an angle of approximately 45° with the central axis of the bit, rather than lying perpendicular to it as in qualified planar fracture. This orientation corresponds to the plane of maximum principal tensile stress under torsion, implying that tensile stress—rather than the shear stress that governs planar fracture—dominates crack propagation [18].

Figure 8b shows the high-magnification microstructure of the matrix region, which exhibits typical mixed-mode characteristics: numerous fine shear dimples and interlaced tear ridges represent ductile behavior through microvoid nucleation, growth, and coalescence, while scattered small cleavage steps and quasi-cleavage facets among the ductile features indicate local brittle fracture. The coexistence of ductile dimple–tear-ridge structures and brittle cleavage features demonstrates that the non-planar fracture follows a mixed ductile–brittle mechanism.

**Figure 9:**
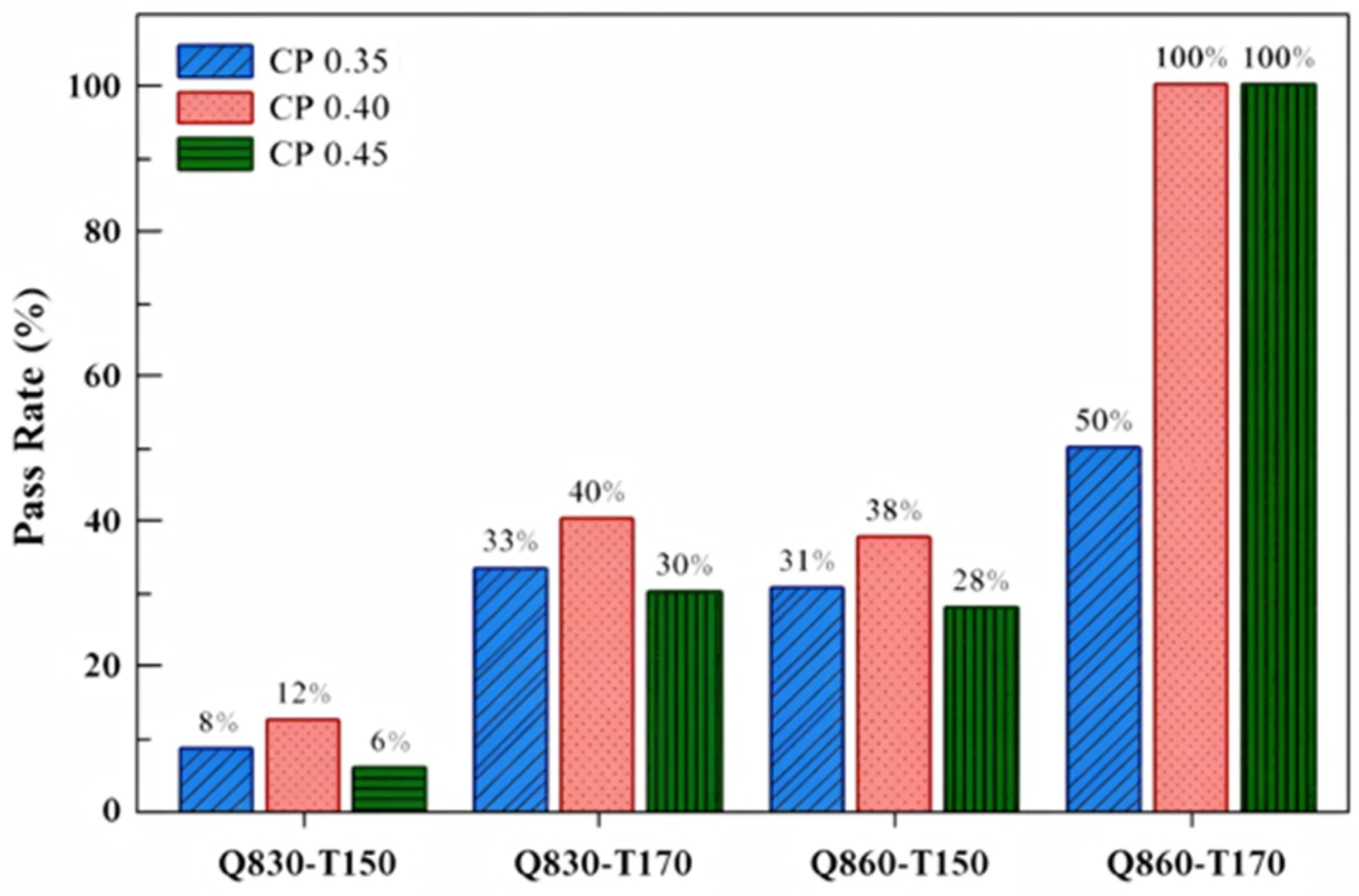
Pass rate of heat-treated specimens under different quenching–tempering processes and carbon potentials (CPs). Elevating the quenching temperature (from 830 °C to 860 °C) and tempering temperature (from 150 °C to 170 °C) both exert a positive effect on the pass rate, and the Q860-T170 process achieves the optimal performance among all groups. For processes with relatively low quenching or tempering parameters (Q830-T150, Q830-T170, Q860-T150), the pass rate first increases and then decreases with the rise in carbon potential, reaching the peak at CP 0.40. For the Q860-T170 process, the pass rate jumps from 50% to 100% when carbon potential increases from 0.35 to 0.40 and remains at 100% under CP 0.45.

Figure 9 summarizes the qualification rate under all tested process conditions. Two clear trends can be identified.

First, both the quenching temperature and tempering temperature exert significant positive effects on the qualification rate. At the same carbon potential, the qualification rate follows the order Q860-T170 > Q860-T150 > Q830-T170 > Q830-T150. This trend directly correlates with the degree of martensite uniformity and the level of residual stress, as will be demonstrated in Section 3.4 and Section 3.5.

Second, carbon potential shows a process-dependent effect. For the optimal Q860-T170 process, the qualification rate increases from 50% at CP 0.35 to 100% at CP 0.40 and remains at 100% at CP 0.45. For other processes with lower quenching or tempering parameters, the qualification rate generally peaks at CP 0.40 and slightly decreases at CP 0.45, suggesting that excessive carbon potential may introduce other microstructural issues when the base process is not fully optimized.

The two failure mechanisms—non-uniform martensite caused by insufficient quenching temperature and surface decarburization caused by low carbon potential—are independent but synergistic. At an 830 °C quenching, even with adequate carbon potential, the non-uniform martensitic microstructure creates internal mechanical heterogeneity that leads to non-planar fracture. At low carbon potential (0.35), even with optimal 860 °C quenching, surface decarburization creates soft ferrite spots that initiate cracks. Only when both factors are properly controlled—860 °C quenching for uniform martensite and 0.40–0.45 carbon potential to prevent decarburization—can a 100% qualification rate be achieved.

### 3.4. Numerical Simulation of Microstructure Uniformity

To quantitatively verify the effect of quenching temperature on martensite uniformity, the quenching process was simulated using Deform-3D. A simplified phase-transformation model, given in Equation (1), was used to describe the austenitization process [19]:

(1) $$\xi_{A}=1-\exp\left\{A{\left[\frac{T-A_{C1}}{A_{C3}-A_{c1}}\right]}^{D}\right\}$$

where $\xi_{A}$ is the volume fraction of austenite; A and *D* are material constants determined by calculation as −5.4 and 2, respectively; *T* is the temperature of the workpiece; $A_{C1}$ is the austenite initiation temperature of 736.75 °C; and $A_{C3}$ is the temperature for complete austenitization at 767.61 °C.

The diffusionless martensitic transformation was described by the Koistinen–Marburger model, given in Equation (2) [20,21,22]:

(2) $$\varepsilon_{M}=1-\exp\left\{\varphi_{1}T+\varphi_{2}\left(C-C_{0}\right)+\varphi_{31}\sigma_{m}+\varphi_{32}\bar{\sigma }+\varphi_{4}\right\}$$

where $\varepsilon_{M}$ is the transformed fraction of martensite; *T* is the instantaneous temperature; $C,C_{0}$ are the current and initial carbon contents; $\sigma_{m}$, $\bar{\sigma }$ is the mean external stress and principal stress; and $\varphi_{1},\varphi_{2},\varphi_{31},\varphi_{32},\varphi_{4}$ are proportional constants.

Because the carbon content remains unchanged and no external stress is applied, only $\varphi_{1}{,\varphi }_{4}$ need to be solved. The values $\varphi_{1}{=0.01833,\varphi }_{4}=-4.6411$ are obtained from the T.T.T. curve.

**Figure 10:**
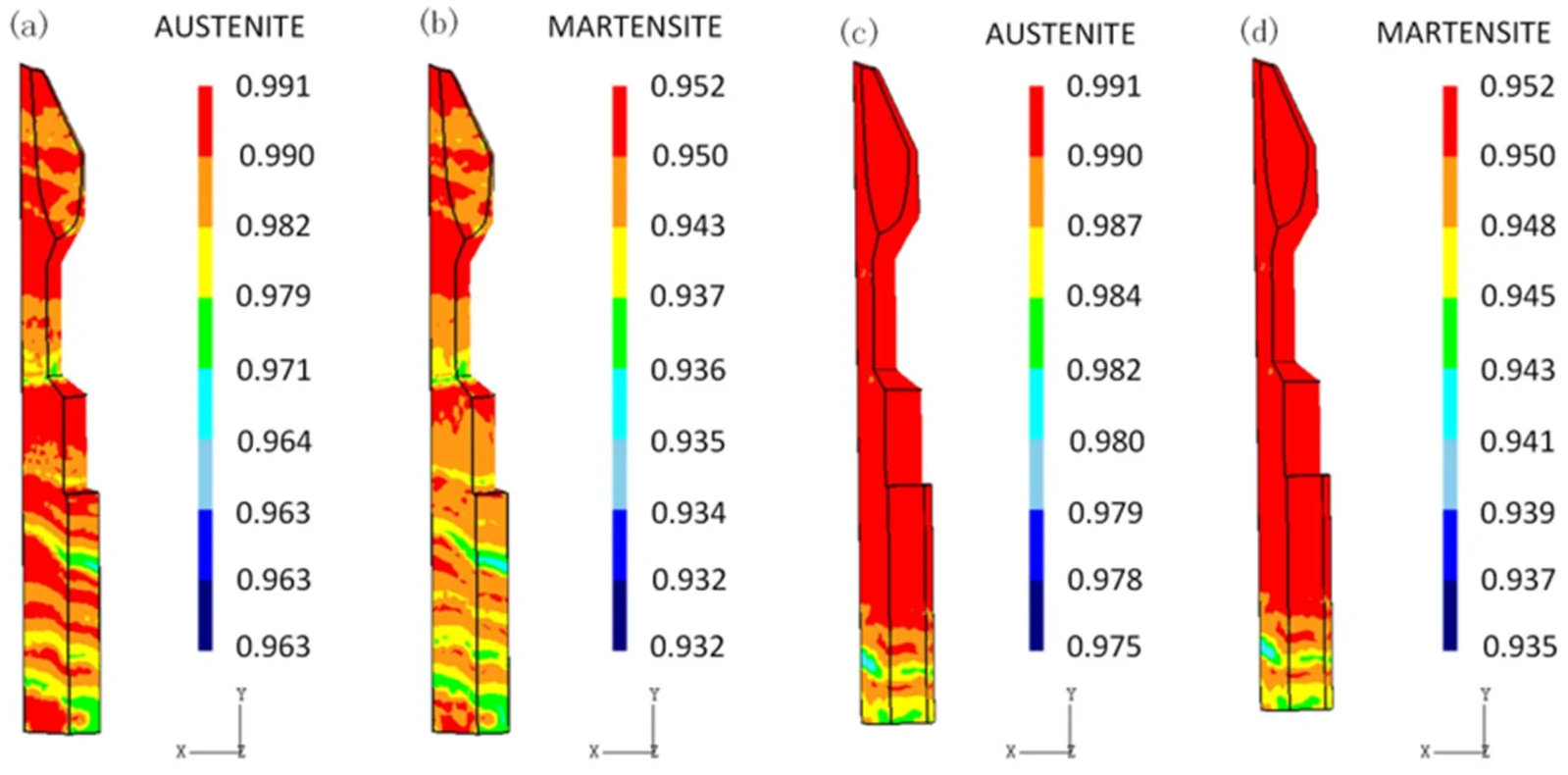
Phase-distribution field after quenching: (**a**) austenite at a quenching temperature of 830 °C; (**b**) martensite at a quenching temperature of 830 °C; (**c**) austenite at a quenching temperature of 860 °C; (**d**) martensite at a quenching temperature of 860 °C.

Figure 10 presents the phase-distribution fields computed at the two quenching temperatures. At an austenitizing temperature of 830 °C (Figure 10a), the austenite volume fraction ranges from 0.982 to 0.991 and is distributed in an obvious layered pattern across the cross-section. This inhomogeneity stems mainly from the insufficient austenitizing temperature: at 830 °C the diffusion of carbon and alloying elements is kinetically limited, so the alloy carbides cannot fully dissolve into the matrix, and the surface-to-core temperature gradient during heating causes asynchronous transformation at different positions. The uneven austenite composition directly affects the subsequent martensitic transformation. As shown in Figure 10b, the martensite formed after cooling is scattered and disordered, with pronounced fluctuations in local volume fraction; regions of insufficient austenitization retain more untransformed structure or form non-martensitic transformation products. This non-uniform martensite distribution directly explains why the 830 °C quenching produces inhomogeneous hardness and a low qualification rate: the bit cannot obtain a 100% martensitic structure, and after tempering it cannot obtain 100% uniform tempered martensite, resulting in extremely uneven internal stress and hardness distribution.

When the quenching temperature is raised to 860 °C, the degree of austenitization improves markedly. As shown in Figure 10c, the austenite volume fraction exceeds 0.99 and is distributed uniformly across the cross-section, with no obvious layered segregation. The higher temperature enhances the diffusion mobility of the carbon and alloying elements, promotes full dissolution of the alloy carbides, and drives the homogenization of the austenite composition. Under identical quenching medium and cooling boundary conditions, uniform austenite of consistent composition has a nearly identical transformation driving force and martensite start temperature throughout the workpiece. The martensitic transformation therefore proceeds synchronously in every region, and the final martensite volume fraction reaches above 0.95 with a highly uniform distribution (Figure 10d). This uniform martensite ensures consistent hardness and mechanical properties from surface to core, providing a favorable microstructural basis for excellent torsional fracture resistance and a qualified planar fracture morphology after tempering.

The simulation results directly confirm the metallographic observations: 860 °C quenching produces uniform martensite, while 830 °C quenching produces non-uniform martensite. This microstructural difference is the fundamental reason why 830 °C quenching exhibits a high failure rate and a low qualification rate regardless of carbon potential level.

### 3.5. Residual Stress and Working Stress

Beyond microstructure uniformity, the multi-level stress fields generated during heat treatment and service also play critical roles in determining the final fracture behavior. During rapid quench cooling, the specimen experiences non-uniform temperature gradients across its cross-section together with the austenite-to-martensite transformation and its attendant volume expansion, which jointly generate thermal stress and transformation stress. Under the coupled superposition of these two components, pronounced stress concentrations appear at three typical regions—the tooth core, the tooth root, and the tooth edge. The transformation stress distribution directly reflects the microstructural evolution during quenching: non-uniform martensitic transformation at 830 °C would produce even more heterogeneous and higher-magnitude transformation stress due to asynchronous volume expansion at different locations.

**Figure 11:**
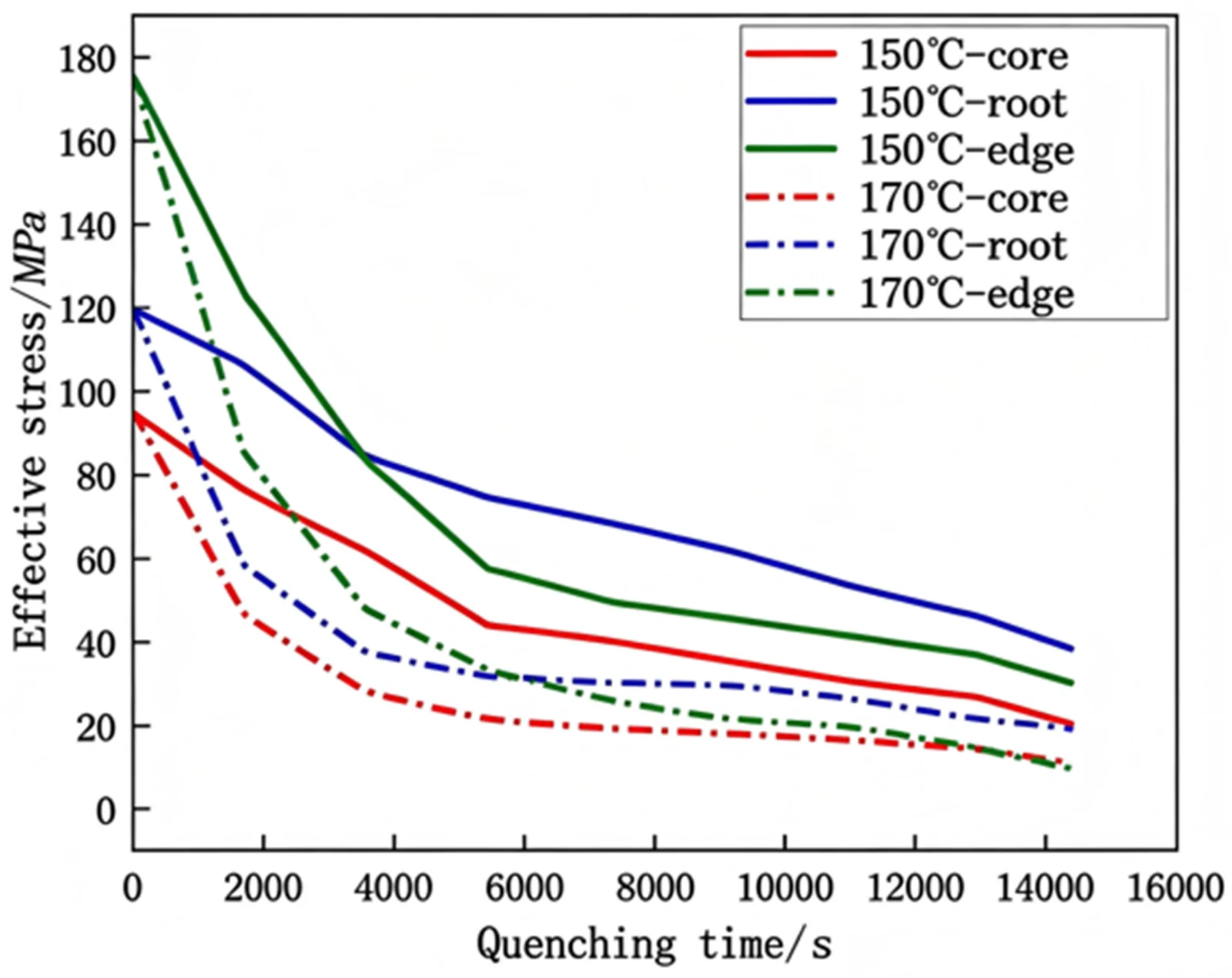
Residual stress in the core, edge, and root of the screwdriver head at different tempering temperatures.

After tempering, the transformation stress is partially relaxed but remains as residual stress. Figure 11 presents the residual stress at the core, edge, and root of the screwdriver head under different tempering temperatures. It can be clearly seen that the residual stress at 150 °C tempering is consistently higher than that at 170 °C across all three positions. In terms of tempering kinetics, raising the tempering temperature from 150 °C to 170 °C facilitates carbon diffusion in the martensite, accelerates the precipitation of fine transition carbides, and improves dislocation mobility, which jointly produce a more pronounced relaxation of both the macroscopic thermal stress and the microstructural residual stress introduced during rapid quenching [23,24,25].

**Figure 12:**
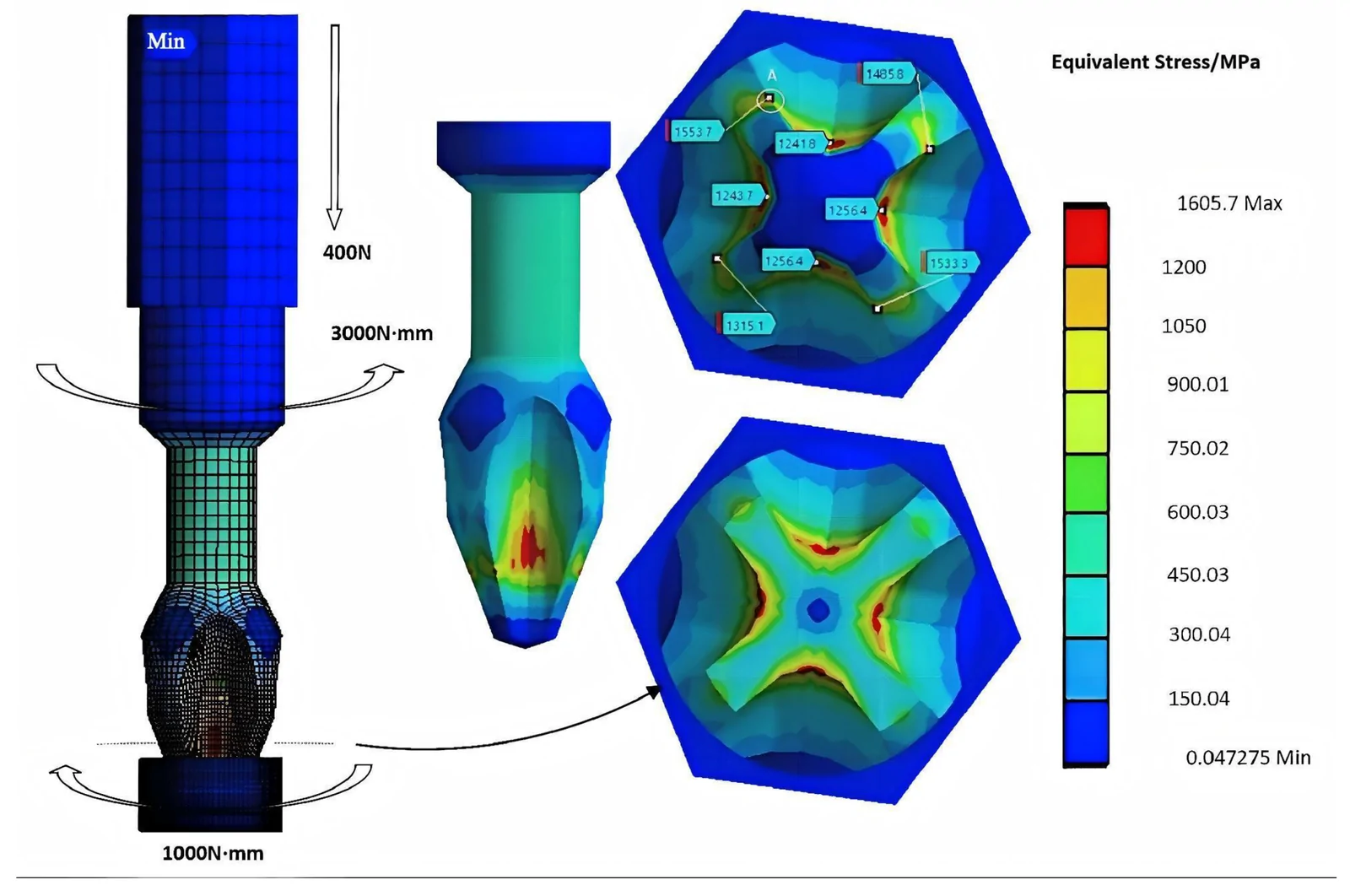
Stress field of the screwdriver head under service loading.

The higher residual stress at 150 °C tempering directly correlates with the lower qualification rate of Q860-T150 compared with Q860-T170. Under cyclic torsional loading, the residual tensile stress superimposes on the applied shear stress, bringing the local equivalent stress closer to the fracture toughness threshold of the material and promoting early crack initiation. Finally, Figure 12 shows the working stress distribution of the screwdriver bit under actual torsional loading, simulated by Ansys Workbench. The simulation reveals severe stress concentration at the tooth edges and tooth roots—the exact locations where fatigue cracks are observed to initiate in fractographic analysis. On the cut section, the equivalent stress increases progressively from the interior toward the outer surface, forming a large stress gradient across the cross-section.

Taken together, the stress analysis reveals a multi-level stress coupling mechanism:

(1) During quenching, non-uniform martensitic transformation (especially at 830 °C) generates heterogeneous transformation stress;
(2) After tempering, insufficient stress relaxation (especially at 150 °C) leaves high residual stress in the working region;
(3) During service, geometric discontinuities at tooth edges and roots create severe working stress concentration.

These three levels of stress—transformation stress, residual stress, and working stress—are superimposed at the same critical locations (tooth edges and roots). When the microstructural basis is sound (uniform tempered martensite, no surface decarburization), the material can withstand this combined stress through uniform ductile deformation, resulting in planar fracture. However, when microstructural defects exist—whether non-uniform martensite from low quenching temperature or soft ferrite from low carbon potential—the local stress concentration at these defect sites exceeds the material’s local strength, triggering multi-source crack initiation and asynchronous propagation that ultimately produces the 45° inclined non-planar fracture.

This integrated analysis establishes a complete failure mechanism chain for S2 steel screwdriver bits:

- Quenching temperature controls bulk microstructure uniformity → 830 °C fails to produce 100% uniform martensite → hardness and stress distribution inhomogeneity → internal crack initiation sites;
- Carbon potential controls surface integrity → CP ≤ 0.35 causes decarburization and massive ferrite → soft surface spots with extreme property mismatch → surface crack initiation sites;
- Tempering temperature controls residual stress level → 150 °C leaves higher residual stress → promotes crack propagation;
- Service loading creates geometric stress concentration → superimposes on residual stress and transformation stress → drives crack growth along maximum tensile stress plane → non-planar fracture.

Only when all four factors are properly controlled—860 °C quenching for uniform martensite, 0.40–0.45 carbon potential to prevent decarburization, 170 °C tempering for residual stress relaxation, and the inherent geometric design maintained—can the bits consistently exhibit planar ductile fracture and achieve a 100% qualification rate.

## 4. Conclusions

Based on systematic experimental characterization and multi-physics numerical simulation, the fracture failure mechanism of S2 alloy steel screwdriver bits was comprehensively elucidated, and an optimized heat-treatment process was developed. The main conclusions are as follows:

(1) Two independent microstructural mechanisms govern the qualification rate of S2 steel screwdriver bits. Quenching temperature determines the bulk microstructure uniformity: at 830 °C, insufficient austenitizing results in non-uniform austenite composition, which transforms into heterogeneous martensite with uneven hardness distribution; at 860 °C, full austenitization produces uniform austenite that transforms into highly uniform martensite (>95% volume fraction) with consistent mechanical properties. Carbon potential controls surface integrity: at CP ≤ 0.35, surface decarburization forms massive ferrite at the near-surface region, creating soft spots with extreme hardness mismatch that act as preferential crack initiation sites; at CP 0.40–0.45, outward carbon diffusion is effectively suppressed and decarburization is eliminated.
(2) The non-planar fracture of screwdriver bits exhibits a mixed ductile–brittle mechanism and follows a multi-source initiation and asynchronous propagation pattern. Cracks initiate from both surface sites (decarburized ferrite regions and tooth-edge stress concentration zones) and internal sites (non-uniform martensite regions and metallurgical defects), then propagate along the maximum principal tensile stress plane at approximately 45° to the bit axis, producing the characteristic uneven, stepped fracture morphology. In contrast, planar fracture under optimized conditions initiates from a single subsurface site at the tooth root and propagates stably along the maximum shear stress plane perpendicular to the axis, exhibiting typical ductile shear dimple features.
(3) A multi-level stress coupling mechanism further amplifies the microstructural effects. During quenching, asynchronous martensitic transformation generates heterogeneous transformation stress. After tempering, insufficient stress relaxation at 150 °C retains high residual stress (consistently higher than at 170 °C across all critical locations). During service, geometric discontinuities at tooth edges and roots create severe working stress concentration. These three stress levels are superimposed at the same critical locations. When microstructural defects exist, the local combined stress exceeds the local material strength, triggering non-planar fracture.
(4) The optimized heat-treatment process—oil quenching after holding at 860 °C for 60 min, followed by air cooling after tempering at 170 °C for 4.0 h, with furnace carbon potential maintained at 0.40–0.45 throughout austenitizing—simultaneously achieves: (a) a uniform tempered martensite microstructure with bulk hardness above 60 HRC, (b) complete elimination of surface decarburization and massive ferrite, (c) effective residual stress relaxation, and (d) consistent mechanical properties across the entire cross-section. This process enables the screwdriver bits to fail by stable planar ductile fracture under torsional load, achieving a 100% qualification rate and providing reliable technical guidance for industrial mass production.

## Figures and Tables

**Table 1:** Nominal chemical composition of the as-received S2 steel.

Element	Content (wt. %)	Element	Content (wt. %)
C	0.45–0.55	V	0.15–0.25
Si	1.00–1.25	Mo	0.40–0.50
Mn	0.45–0.60	Cu	0–0.25
Ni	0.10–0.30	P	≤0.013
Cr	0.20–0.40	S	≤0.003

## Data Availability

The original contributions presented in this study are included in the article/Supplementary Material. Further inquiries can be directed to the corresponding author.

## Supplementary Materials

The following supporting information can be downloaded at https://www.mdpi.com/article/10.3390/ma19163443/s1, S1: Plastic Parameters; S2: Elastic Parameters; S3: Thermophysical Parameters; S4: Transformation Parameters. Figure S1: Flow stress curves of S2 tool steel under different conditions; Figure S2: Material elasticity parameters; Figure S3: Material thermophysical property parameters; Figure S4: Material phase transition parameters.

## References

[1] Duryat R.S. (2020). The screwdriver: A basic review on design and material selection. AIP Conf. Proc..

[2] Tong S.H., Yang D.C.H. (2012). Involute-Based Screw Driver Profiles with High Load Capacity. J. Mech. Des..

[3] Nieoczym A., Drozd K., Veselik P. (2019). Mathematical Model of Energy Processes in an Industrial Electric Screwdriver. Sci. Tech..

[4] Gowd G.H., Vali S.S., Ajay V., Mahesh G.G. (2014). Experimental Investigations & Effects of Cutting Variables on MRR and Tool Wear for AISI S2 Tool Steel. Procedia Mater. Sci..

[5] Momeni M., Kheirandish S., Saghafian H., Hedjazi J., Momeni M. (2014). Effects of heat treatment on mechanical properties of modified cast AISI D3 tool steel. Mater. Des..

[6] Li H., Mi Z., Zhang X., Tang D., Wang Y. (2014). Carbide dissolution during intercritical austenitization in bearing steel. J. Wuhan Univ. Technol.-Mater. Sci. Ed..

[7] Il’ina V.P. (1999). Effect of surface decarburization on the susceptibility of high-strength steel 38Kh5MSFA to brittle fracture. Met. Sci. Heat Treat..

[8] Zhu H., Gao X., Shao Y., Li K., He W., Yu Z. (2025). Strain-controlled torsional fatigue and fracture of EQ56 high-strength steel. J. Constr. Steel Res..

[9] Spriestersbach D., Grad P., Kerscher E. (2014). Influence of different non-metallic inclusion types on the crack initiation in high-strength steels in the VHCF regime. Int. J. Fatigue.

[10] Mackerle J. (2003). Finite element analysis and simulation of quenching and other heat treatment processes: A bibliography (1976–2001). Comput. Mater. Sci..

[11] Esfahani A.K., Babaei M., Sarrami-Foroushani S. (2021). A numerical model coupling phase transformation to predict microstructure evolution and residual stress during quenching of 1045 steel. Math. Comput. Simul..

[12] Kim N.K., Bae K.Y. (2015). Analysis of deformation in the carburizing-quenching heat treatment of helical gears made of SCM415H steel. Int. J. Precis. Eng. Manuf..

[13] Sugianto A., Narazaki M., Kogawara M., Shirayori A., Kim S.Y., Kubota S. (2009). Numerical simulation and experimental verification of carburizing-quenching process of SCr420H steel helical gear. J. Mater. Process. Technol..

[14] Vijayarangan S., Ganesan N. (1994). Static contact stress analysis of a spur gear tooth using the finite element method, including frictional effects. Comput. Struct..

[15] Zhao F., Zhang C., Liu Y. (2016). Ferrite Decarburization of High Silicon Spring Steel in Three Temperature Ranges. Arch. Metall. Mater..

[16] Wang H., Sun S. (2022). A Study of the Mechanism of Formation of Composite Oxide Scale on Heat-Resistant Steel 25Cr18Ni9Si2. Met. Sci. Heat Treat..

[17] Ghajar R., Mirone G., Keshavarz A. (2013). Ductile failure of X100 pipeline steel–Experiments and fractography. Mater. Des..

[18] Murakami Y., Takahashi K. (1998). Torsional fatigue of a medium carbon steel containing an initial small surface crack introduced by tension–compression fatigue: Crack branching, non-propagation and fatigue limit. Fatigue Fract. Eng. Mater. Struct..

[19] Reti T., Fried Z., Felde I. (2001). Computer simulation of steel quenching process using a multi-phase transformation model. Comput. Mater. Sci..

[20] Koistinen D., Marburger R. (1959). A general equation prescribing the extent of the austenite-martensite transformation in pure iron-carbon alloys and plain carbon steels. Acta Metall..

[21] Wilson E., Medina S. (2000). Application of Koistinen and Marburger’s athermal equation for volume fraction of martensite to diffusional transformations obtained on continuous cooling 0·13%C high strength low alloy steel. Mater. Sci. Technol..

[22] Jung M., Kang M., Lee Y.K. (2012). Finite-element simulation of quenching incorporating improved transformation kinetics in a plain medium-carbon steel. Acta Mater..

[23] Torres M. (2002). An evaluation of shot peening, residual stress and stress relaxation on the fatigue life of AISI 4340 steel. Int. J. Fatigue.

[24] Luo X., Ren S., Han S., Hu R. (2025). Effects of tempering mode on the phase transformation and mechanical properties of 30CrMnSiNi2A steel within the tempered martensite embrittlement regime. Mater. Charact..

[25] Zhang Y., Marusawa K., Kudo K., Morooka S., Harjo S., Miyamoto G., Furuhara T. (2024). Multi-aspect Characterization of Low-temperature Tempering Behaviors in High-carbon Martensite. ISIJ Int..

