# Supplementary material for: Simulation Analysis on the Fracture Failure of S2 Alloy Steel Screwdriver Bits
Source: Materials (Basel). 2026 Aug 14;19(16):3443. doi: 10.3390/ma19163443 (PMC13514429; doi:10.3390/ma19163443)
Supplement: Supplementary file 1 [file materials-19-03443-s001.zip › materials-4438963-supplementary.pdf]

Online Resource : Establishment of S2 shock-resistant tool steel Material Database

Simulation Analysis on the Fracture Failure of S2 Alloy Steel Screwdriver Bits

The material library of Deform software does not contain the material model for S2 shock-resistant tool steel. Therefore, thermodynamic calculation adopted in this paper to calculate and revise the material parameters required for heat treatment, so as to construct the corresponding material models. The data to be imported mainly include plastic parameters, elastic parameters, thermophysical parameters and phase transformation parameters of the material.

S1) Plastic Parameters

Plastic parameters mainly refer to the flow stress curves of each phase, which characterize the plastic deformation behavior of metallic microstructures. Metals initiate plastic flow or deformation once the material reaches the yield stress or flow stress. Figures S1 (a to e) present the room-temperature flow stress curves of austenite, ferrite, pearlite, bainite and martensite in S2 alloy tool steel, respectively.

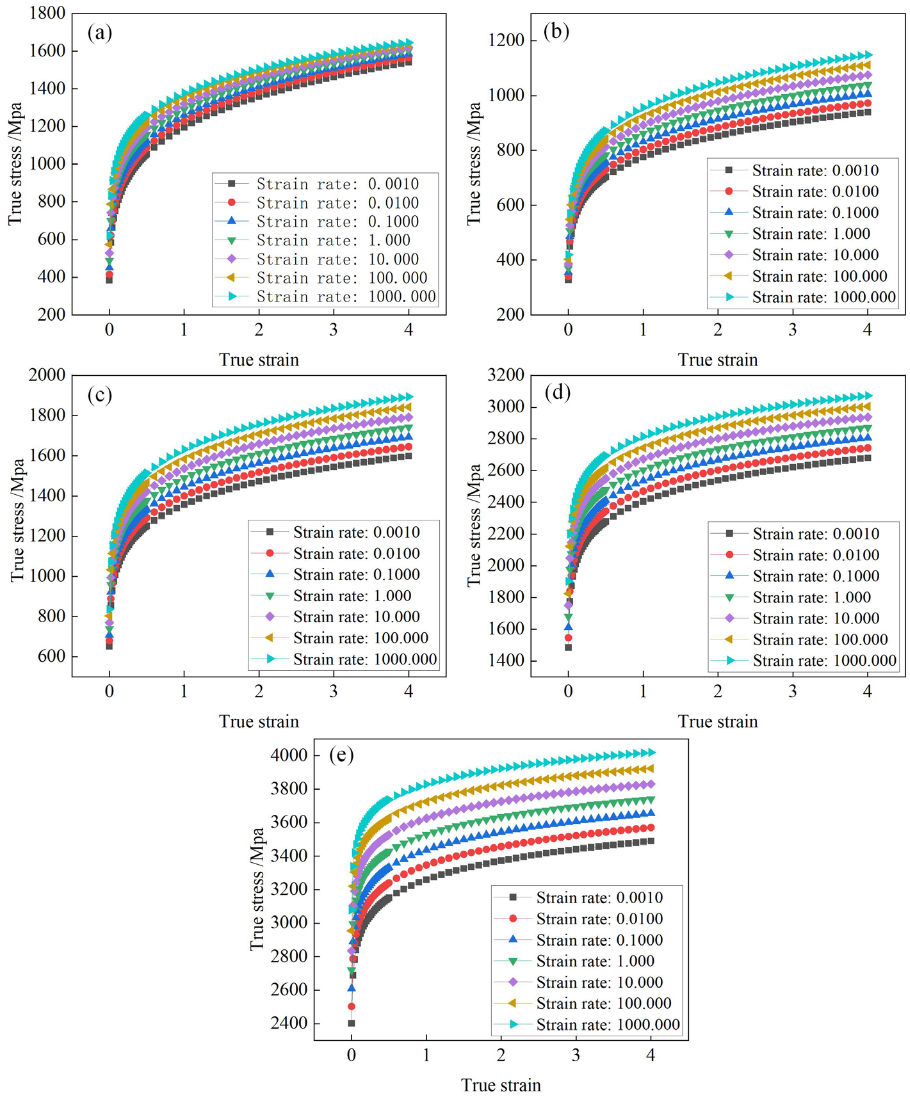

Figure S1 Flow stress curves of S2 tool steel under different conditions
(a) austenite; (b) ferrite; (c) pearlite; (d) bainite; (e) martensitic

S2) Elastic Parameters

The elastic deformation process of metallic materials needs to be described by defining the Young's modulus, Poisson's ratio and thermal expansion coefficient of the base material as well as each constituent phase. The elastic parameters imported in this study are shown in Figure S2. Herein, the thermal expansion coefficient quantifies volumetric strain induced by temperature variation. During thermal expansion, the overall expansion is less governed by the coefficient of the bulk material but strongly dependent on the thermal expansion coefficients of individual phases. Therefore, the bulk thermal expansion coefficient of the material is determined as 1.3×10^{-5} ℃$^{-1}$.

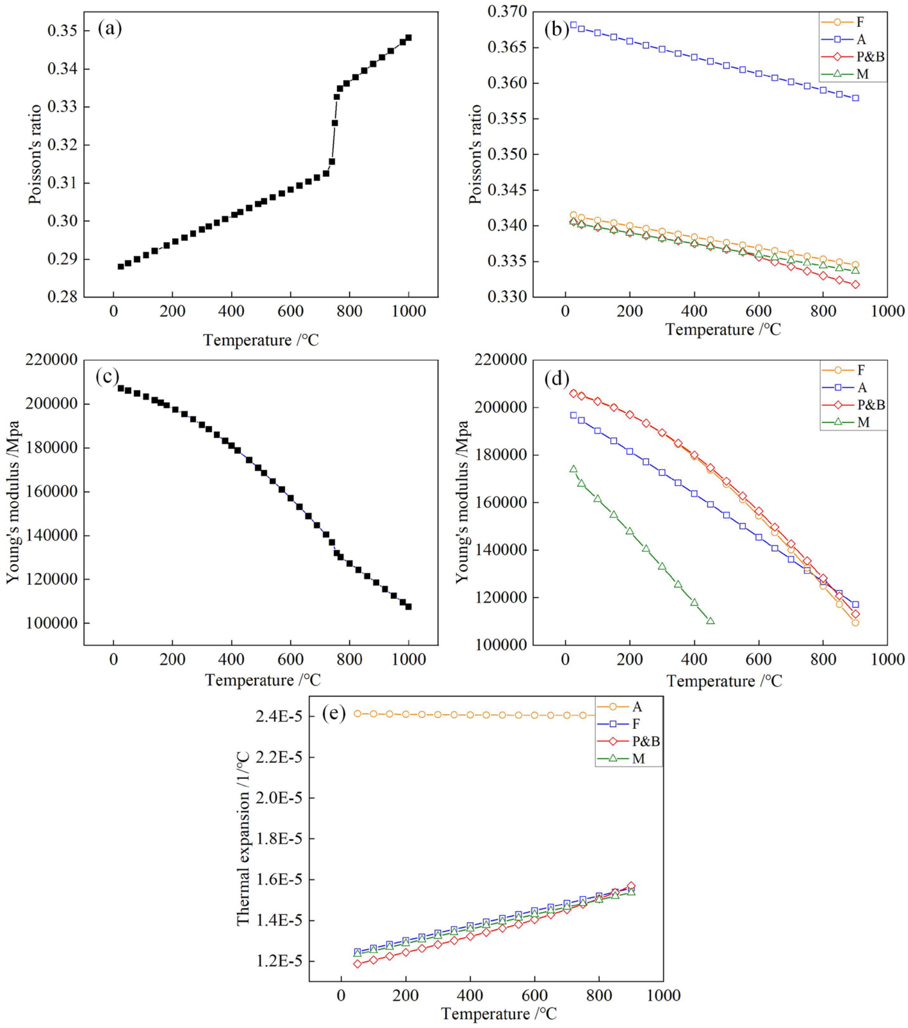

Figure S2 Material elasticity parameters

(a) Poisson's ratio of S2 steel; (b) Poisson's ratio of each phase; (c) Young's modulus of S2 steel;

(d) Young's modulus of each phase;(d) Thermal expansion coefficient of each phase

S3) Thermophysical Parameters

The thermophysical parameters of materials mainly include thermal conductivity and heat capacity. Thermal conductivity reflects the heat transfer capacity of the material. The thermophysical parameters adopted in this paper are displayed in Figure S3.

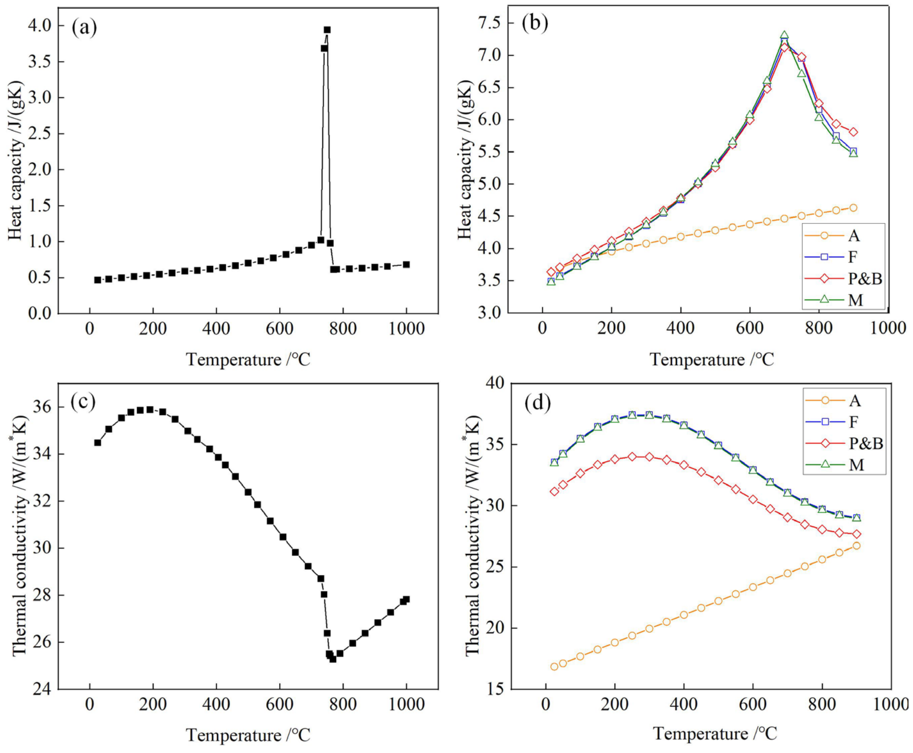

Figure S3 Material thermophysical property parameters

(a) Heat capacity of S2 steel; (b) Heat capacity of each phase;

(c) Thermal conductivity of S2 steel; (d) Thermal conductivity of each phase

S4) Transformation Parameters

Phase transformation parameters mainly cover latent heat of phase transformation, volume change during phase transformation, and selection of phase transformation kinetic models. The latent heat and volume variation of phase transformation between various phases in S2 alloy steel are presented in Figure S4.

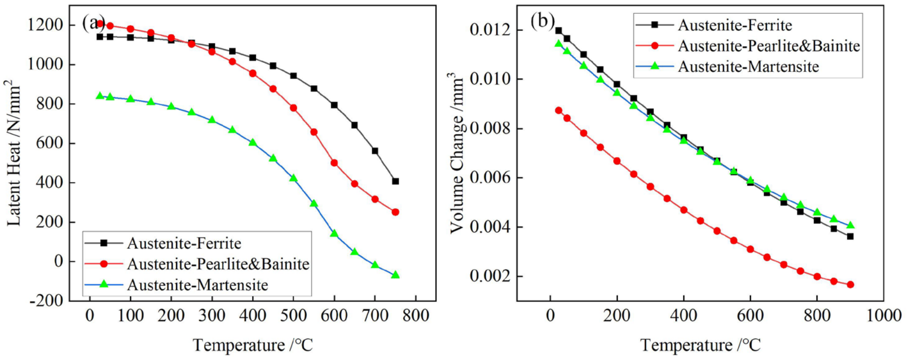

Figure S4 Material phase transition parameters

(a) Latent heat of phase transition; (b) Volume change of phase transition
